# Predictive models for intrapartum maternal fever: Development and validation of pre-analgesia and labor process indicators

**DOI:** 10.1097/MD.0000000000042939

**Published:** 2025-06-20

**Authors:** Bo Liu, Liang Ling, Dayuan Wei, Yuanling Li, Fei Jia, Huiru Li, Na Li, Hongquan Xiao, Jian Zhang

**Affiliations:** a Department of Anesthesiology, Chengdu Jinjiang District Women & Children Health Hospital, Chengdu, Sichuan, China; b Department of Anesthesiology, Sichuan Women’s and Children’s Hospital/Women’s and Children’s Hospital, Chengdu Medical College, Chengdu, Sichuan, China.

**Keywords:** intrapartum fever, labor analgesia, optimal predictors, predictive model

## Abstract

Combined spinal–epidural anesthesia is effective for labor pain relief but is associated with increased rates of intrapartum maternal fever, which can negatively impact maternal and neonatal outcomes. This study aimed to develop and validate 2 predictive models: one to assess the risk of fever before labor analgesia (model B) and another to evaluate the risk of fever throughout the labor process (model W). This retrospective case-control study was conducted at Chengdu Jinjiang District Maternal & Child Health Hospital, including 2783 parturients who received labor analgesia between January 2021 and March 2022. Stepwise logistic regression was used to identify clinical predictive indicators, followed by multivariate logistic regression to determine intrapartum fever predictors. Model performance was assessed using the Hosmer–Lemeshow test and areas under the receiver operating characteristic curves (AUROCs). A total of 2276 patients were included in the development cohort and 507 in the validation cohort. Optimal predictors for model B included primiparity, neutrophil count, anemia, estimated fetal weight, body surface area, and cervical dilation before analgesia. For model W, predictors included height, primiparity, anemia, neutrophil count, estimated fetal weight, total duration of labor, and time from rupture of membranes to delivery. AUROCs for models B and W were 0.698 and 0.740, respectively; external validation showed AUROCs of 0.703 and 0.797. In conclusion, model B effectively predicts fever risk before labor analgesia, though its predictive efficiency is lower than model W, which better predicts fever risk after analgesia. The combination of these 2 models will aid in the early identification and management of high-risk parturients, thereby reducing the incidence of intrapartum fever and improving maternal and neonatal outcomes.

## 1. Introduction

Labor analgesia significantly alleviates the pain experienced by parturients during childbirth.^[1]^ However, most studies suggest that labor analgesia is closely related to an increased incidence of intrapartum fever.^[2]^ Intrapartum fever may increase the likelihood of women undergoing cesarean delivery and could lead to an increased incidence of neonatal encephalopathy, along with a series of adverse reactions.^[3–7]^ The mechanisms of intrapartum fever are still being explored. In the face of a febrile parturient, obstetricians are more inclined to perform a cesarean delivery. Therefore, it is extremely important to identify high-risk febrile parturients and take specific measures to reduce the rate of fever.^[8–11]^

Currently, several predictive models have been applied to the prediction of intrapartum fever. However, the current predictive models incorporate indicators from the entire labor process, such as primiparity, fetal weight, the second stage of labor, and the pulse perfusion index at different time points. Although these models demonstrate good predictive performance, most of the indicators are obtained postpartum.^[12,13]^ Compare the predictive performance of a model constructed solely from indicators available before labor analgesia with that of a model developed using indicators from the entire labor process. Our aim is to establish and validate 2 predictive models: one that utilizes factors available before labor analgesia and another that incorporates factors from the entire labor process. Additionally, we explore the foundation they provide for clinical decision-making at different stages of labor.

## 2. Methods

### 2.1. Study design and participants

This study was approved by the Ethics Committee of Chengdu Jinjiang District Women & Children Health Hospital (approval number: 2024 trial (20)) and adopted a retrospective cohort study design. Owing to the retrospective design of the study, the requirement for written informed consent was waived by both ethics committees. A total of 2899 parturients who received combined spinal–epidural anesthesia from January 2021 to March 2022 were selected from the electronic medical record system according to the following exclusion criteria: a prenatal diagnosis of infectious diseases, long-term steroid drug use or a prenatal history of nonsteroidal antipyretic analgesic use, a basal body temperature higher than 37.2°C and incomplete clinical data.

### 2.2. Data collection

The baseline data of the patients were collected through an electronic medical record system, including age, height, weight, body mass index, body surface area, and gestational week. Additionally, information on comorbidities such as gestational diabetes mellitus (GDM), pregnancy-induced hypertension, and anemia was collected, along with admission laboratory test results. Furthermore, relevant indicators prior to labor analgesia were recorded, including the use of oxytocin, the number of vaginal examinations, and the degree of cervical dilation before analgesia. Throughout the entire labor process, indicators such as the duration of labor analgesia, total duration of labor, time from the rupture of membranes to delivery, use of oxytocin, and the number of vaginal examinations were also collected.

In this study, the index before labor analgesia refers to the clinical index that could be determined before labor analgesia, and the index of the whole labor and childbirth process refers to the clinical index that could be obtained during the whole labor and childbirth process (including the clinical index before and after labor analgesia).

### 2.3. Outcomes

The primary outcome of this study was the predictive ability of the models in assessing the risk of intrapartum maternal fever, quantified by the AUCROC. A higher AUC indicates better discrimination capability of the models. The secondary outcomes included the calibration of the models, assessed through calibration plots to evaluate the agreement between predicted probabilities and observed outcomes. Additionally, decision curve analysis (DCA) was conducted to assess the practical utility and net benefit of the models in clinical application.

### 2.4. Statistical analysis

SPSS 25.0 software and R 4.3.1 software were used for statistical analysis. Normally distributed measurement data are expressed as the mean ± standard deviation and were compared using the independent-sample *t* test. Measurement data with a nonnormal distribution are expressed as the median and interquartile range. Categorical data are presented as percentages (%), and comparisons between groups were performed by the *χ*^2^ test. Univariate analysis was used for the preliminary selection of candidate factors. By stepwise logistic regression analysis, clinical indicators were selected and included in multivariate logistic regression to determine the predictive factors of intrapartum fever, and retention in the logistic regression model required a *P* < .05.

The prediction model was developed according to the results of multivariate logistic regression analysis. The receiver operating characteristic (ROC) curve was drawn, the areas under the receiver operating characteristic curve (AUROC) was used to evaluate the discrimination of the model, and the Hosmer–Lemeshow goodness-of-fit test was used to evaluate model calibration. *P* > .05 indicated that the calibration of the model was good. At the same time, the visual nomogram was drawn by R language to display the final model, and the ROC curve, AUROC, DCA were used to evaluate and validate the performance of the nomogram model. Using the data of the validation cohort, the prediction probability was calculated strictly according to the original model, the ROC curve was drawn, and the AUROC was calculated for external verification of the model.

This prediction model includes 30 predictors, each requiring at least 10 participants.^[14]^ Therefore, we need at least (30 × 10/0.2) = 1500 participants.

## 3. Results

**Figure 1.**
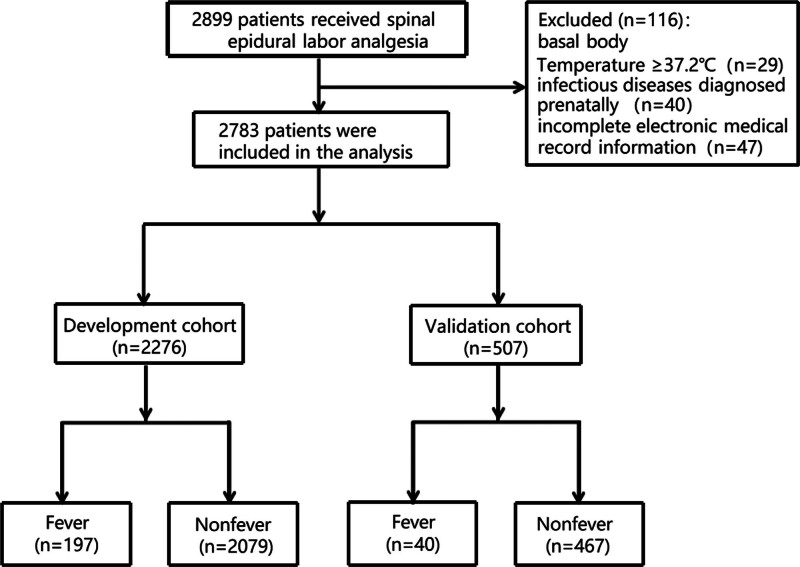
Participant recruitment flowchart.

A total of 2899 parturients were initially enrolled in this study. Twenty-nine parturients were excluded due to a prenatal body temperature ≥ 37.2°C. Fourty parturients were excluded due to prenatal infectious disease diagnoses, 47 parturients were excluded due to incomplete electronic medical record information, and 2783 parturients were eligible for the study; 2276 parturients were included in the development cohort, and 507 were included in the validation cohort (Fig. 1). The incidence of fever was 8.66% (197/2276) in the development cohort and 7.89% (40/507) in the validation cohort. The comparison of maternal data between the development cohort and the validation cohort showed no significant differences (*P* > .05) (Table S1, Supplemental Digital Content, http://links.lww.com/MD/P226).

### 3.1. Univariate analysis of the 2 groups (development cohort)

In the development cohort, univariate analysis showed that the values were significantly higher for the parturients with intrapartum fever than for those without intrapartum fever for the white blood cell count, neutrophil (NEUT) count, neutrophil-to-lymphocyte ratio, proportion of primiparas, proportion of parturients with anemia, number of vaginal examinations during the whole labor process and estimated fetal weight (*P* < .05) (Table 1). Furthermore, the parturients with intrapartum fever more commonly used oxytocin during the whole labor process; the duration of labor analgesia, total duration of labor, and time from the rupture of membranes to delivery were significantly longer; and the cervical dilatation degree before labor analgesia was significantly smaller (*P* < .05) (Table 1).

**Table 1:** Univariate analysis of the 2 groups (development cohort).

Variable	Fever (n = 197)	Non-fever (n = 2079)	*P* value
Age (yr)	28.44 ± 3.64	28.59 ± 3.75	.583
Height (cm)	158.80 ± 4.83	159.40 ± 4.70	.088
Weight (kg)	66.07 ± 8.53	65.89 ± 8.05	.762
BMI (kg/m^2^)	26.17 ± 2.97	25.93 ± 2.90	.248
Body surface area (m^2^)	1.79 ± 0.13	1.9 ± 0.12	.825
Gestational age (w)	39.46 ± 1.07	39.30 ± 1.27	.107
WBC count (10^9^/L)	9.56 ± 2.93	9.12 ± 2.41	.044
LYM count (10^9^/L)	1.50 ± 0.43	1.52 ± 0.42	.537
LYM (%)	16.95 ± 5.61	17.39 ± 5.41	.276
NEUT count (10^9^/L)	7.33 ± 2.75	6.88 ± 2.23	.026
NEUT (%)	75.00 ± 6.84	74.59 ± 6.9	.382
PLR (%)	125.25 ± 48.15	120.53 ± 47.06	.179
NLR (%)	5.24 ± 2.45	4.89 ± 2.24	.038
Estimated fetal weight (g)	3297.97 ± 358.31	3196.27 ± 369.23	<.001
Primiparity	189 (95.9%)	1671 (80.4%)	<.001
GDM	42 (21.3%)	484 (23.3%)	.596
Hypertension during pregnancy	7 (3.6%)	83 (4.0%)	1.000
Anemia	65 (33.0%)	427 (20.5%)	<.001
Hepatitis B	11 (5.6%)	102 (4.9%)	.609
Hypothyroidism	13 (6.6%)	124 (6.0%)	.753
PROM	71 (36.2%)	715 (34.4%)	.639
Cervical dilatation degree before labor analgesia (cm)	1.2 ± 0.6	1.4 ± 0.9	<.001
Number of vaginal examinations before labor analgesia	2.6 ± 1.3	2.5 ± 1.2	.283
Oxytocin use before labor analgesia	72 (36.5%)	726 (34.9%)	.640
Number of vaginal examinations*	6.44 ± 2.37	5.71 ± 2.25	<.001
Oxytocin*	114 (57.9%)	1012 (48.7%)	.014
Amniotic fluid pollution III*	39 (19.8%)	336 (16.2%)	.192
Duration of labor analgesia (min)*	582.72 ± 265.35	425.70 ± 278.25	<.001
Total duration of labor (min)*	761.94 ± 269.61	593.70 ± 265.21	<.001
Time from the rupture of membranes to delivery (min)*	537.40 ± 400.16	384.02 ± 385.32	<.001

BMI = body mass index, DCA = decision curve analysis, GMD = gestational diabetes mellitus, LYM = lymphocyte, NEUT = neutrophil, NLR = neutrophil-to-lymphocyte ratio, PLR = platelet-to-lymphocyte ratio, PROM = premature rupture of membranes, WBC = white blood cell.

* The index of the whole labor and childbirth process.

### 3.2. Multivariate logistic regression analysis

**Figure 2.**
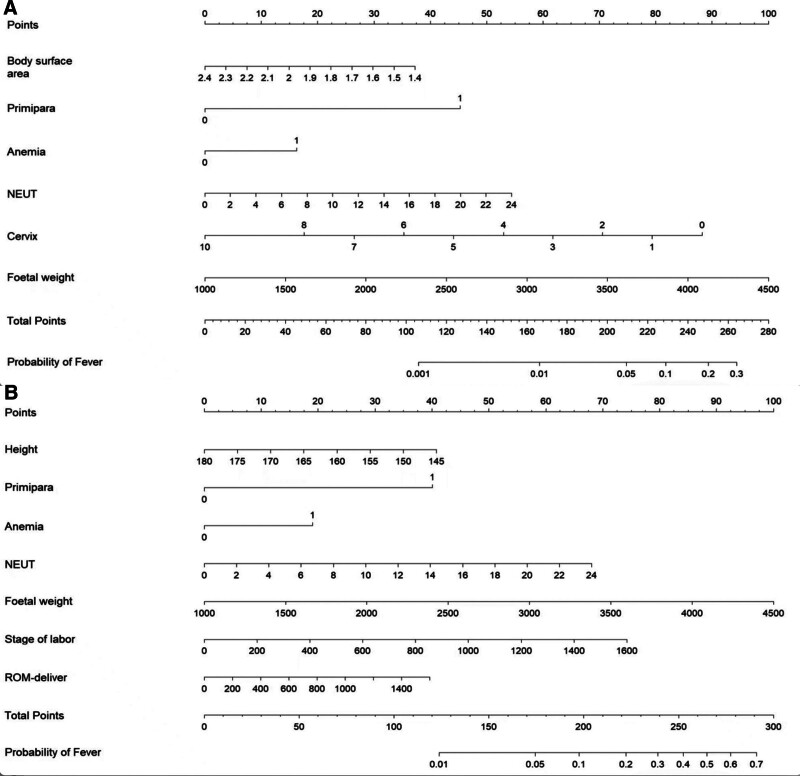
(A) The nomogram for model B incorporates body surface area, primiparity, NEUT count, anemia, cervical dilation before labor analgesia, and estimated fetal weight. (B) The nomogram for model W includes height, primiparity, NEUT count, anemia, estimated fetal weight prior to delivery, total duration of labor, and the time from rupture of membranes to delivery. To utilize the nomogram, locate the individual patient’s value on each variable axis and draw a line upward to determine the points allocated for each variable. The sum of these points is then found on the total points axis, from which a line can be drawn downward to the risk of fever axis to ascertain the likelihood of developing a fever. NEUT = neutrophil.

In the development cohort, multivariate logistic regression analysis was conducted for predictive models B and W, employing stepwise logistic regression for model W. The final selected factors for model B included body surface area, primiparity, NEUT count, anemia, cervical dilation degree prior to labor analgesia, and estimated fetal weight, all of which were incorporated into the multiple-factor logistic regression analysis. For model W, the factors included height, primiparity, anemia, NEUT count, estimated fetal weight, total duration of labor, and time from membrane rupture to delivery, which were also analyzed using multiple-factor logistic regression. The results indicated that in model B, primiparity, a high NEUT count, anemia, and greater estimated fetal weight were identified as risk factors for intrapartum fever. Conversely, a larger body surface area and a smaller cervical dilation degree prior to labor analgesia served as protective factors against intrapartum fever (Table 2). In model W, primiparity, NEUT count, anemia, estimated fetal weight, total duration of labor, and time from membrane rupture to delivery were categorized as risk factors for intrapartum fever, while height was identified as a protective factor (Table 3). Based on logistic regression analysis, models B and W were constructed and represented as nomograms (Fig. 2).

**Table 2:** Multivariate logistic regression analysis of the 2 groups (model B).

Variable	β	SE	Wals	OR	95% CI	*P* value
Body surface area	‐1.421	0.689	4.361	0.242	0.064–0.916	.037
Primiparity	1.735	0.368	22.187	5.669	2.754–11.670	<.001
NEUT count	0.087	0.031	7.697	1.091	1.026–1.160	.006
Anemia	0.623	0.165	14.325	1.865	1.351–2.576	<.001
Cervical dilatation degree before labor analgesia	‐0.338	0.123	7.543	0.713	0.560–0.908	.006
Estimated fetal weight	0.001	0.000	21.142	1.001	1.001–1.002	<.001

NEUT = neutrophil.

**Table 3:** Multivariate logistic regression analysis of the 2 groups (model W).

Variable	β	SE	Wals	OR	95% CI	*P* value
Height	‐0.038	0.017	5.087	0.963	0.932–0.995	.024
Primiparity	1.304	0.378	11.866	3.683	1.754–7.733	.001
NEUT count	0.092	0.032	8.194	1.097	1.030–1.168	.004
Anemia	0.620	0.168	13.640	1.859	1.338–2.583	<.001
Estimated fetal weight	0.001	0.000	15.888	1.001	1.000–1.001	<.001
Total duration of labor*	0.002	0.000	28.625	1.002	1.001–1.002	<.001
Time from the rupture of membranes to delivery*	0.001	0.000	18.524	1.001	1.000–1.001	<.001

NEUT = neutrophil.

* The index of the whole labor and childbirth process.

### 3.3. Calibration plots and DCA

**Figure 3.**
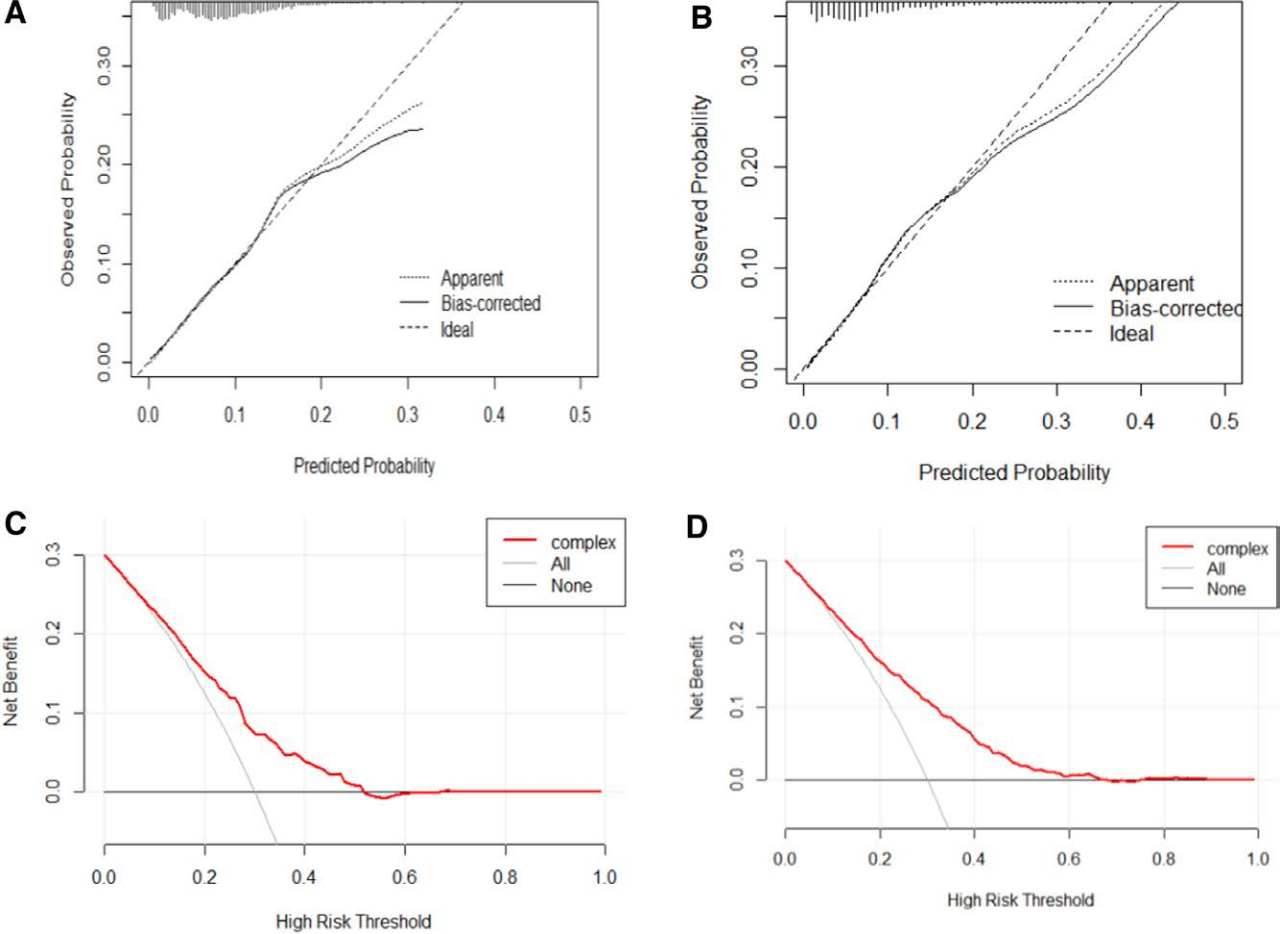
(A) The calibration plots of model B. (B) The calibration plots of model W. (C) DCA of model B. (D) DCA of model W. DCA = decision curve analysis.

The calibration plots of the models showed a strong correspondence between the predicted probabilities of intrapartum fever and the actual observed probabilities (Fig. 3A, B). Additionally, the DCA demonstrated that both models provided higher net clinical benefits at various decision thresholds (Fig. 3C, D).

### 3.4. Evaluation of model accuracy and external validation

**Figure 4.**
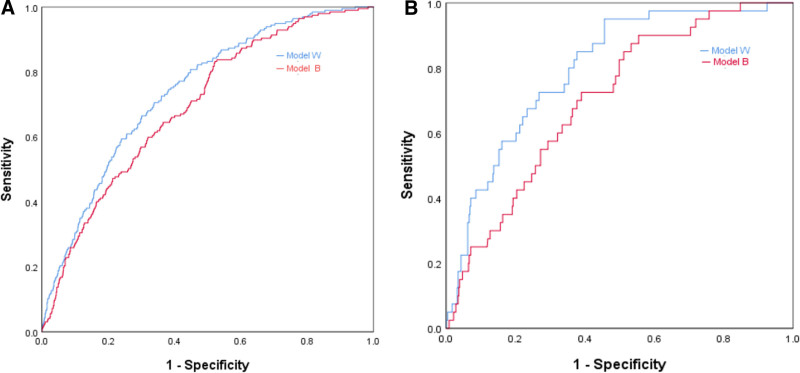
(A) ROC curve of the development cohort. (B) ROC curve of the validation cohort (model B). ROC = receiver operating characteristic.

ROC curves were utilized to assess the accuracy of the models. For model B, the AUROC was 0.696 (95% CI: 0.660–0.732), with a maximum cutoff value of 0.312, a sensitivity of 83.2%, and a specificity of 47.9%. The Hosmer–Lemeshow goodness-of-fit test yielded a statistic of 4.026 (*P* = .855), indicating a good level of agreement between the predicted and observed values. In contrast, model W demonstrated an AUROC of 0.740 (95% CI: 0.706–0.773), with a maximum cutoff value of 0.368, a sensitivity of 66.2%, and a specificity of 70.6%. The Hosmer–Lemeshow goodness-of-fit test for model W resulted in a statistic of 4.371 (*P* = .800) (Fig. 4A).

The study included a total of 507 parturients who received combined spinal–epidural anesthesia from January to March 2022. Among these, 40 parturients (7.89%) were classified in the fever group, while 467 (92.11%) were in the non-fever group. External validation of models B and W was performed using R software, revealing an AUROC of 0.703 (95% CI: 0.629–0.777) for model B and 0.797 (95% CI: 0.733–0.861) for model W (Fig. 4B).

## 4. Discussion

In this retrospective study, we systematically analyzed the risk factors for intrapartum fever both before labor analgesia and throughout the entire labor process. We successfully established 2 predictive models for assessing the risk of intrapartum fever. Our findings revealed that the AUROCs of models B and W were 0.696 and 0.740, respectively, indicating superior predictive performance for model W. Although model B demonstrated a relatively weaker predictive capability compared to model W, it nonetheless exhibited significant potential in identifying parturients at risk of fever prior to the administration of labor analgesia. This early identification can enable timely interventions and enhance patient management, making model B a valuable tool for clinicians, particularly anesthesiologists, looking to mitigate the risks associated with intrapartum fever.

Considering the potential adverse effects of intrapartum fever on both the parturient and the newborn, the clinical community has been striving to identify parturients at high risk for fever through predictive models. However, current predictive models have not been able to forecast whether a parturient will develop fever in advance. Our established model B can, to some extent, assist clinicians in predicting the risk of fever using commonly available clinical indicators, thereby identifying at-risk parturients and implementing early interventions to reduce the potential risk of fever. This model offers a new possibility for enhancing the safety of both parturients and newborns, demonstrating its potential application in clinical practice.

### 4.1. Predictive model using pre-analgesia factors

Primiparity, NEUT count, anemia, and estimated fetal weight were optimal predictors for the model, which was consistent with previous studies.^[12,15,16]^ The new predictive factors found in this study were body surface area and the cervical dilatation degree before labor analgesia. The AUROC of model B was 0.696, and the calibration plots also indicated good calibration.

The degree of cervical dilation before labor analgesia is inversely related to the duration of labor analgesia experienced by parturients. Research has indicated that this duration is closely linked to the occurrence of fever.^[17]^ Additionally, body surface area plays a crucial role in heat dissipation; parturients with a larger body surface area can exchange heat with their environment more efficiently, resulting in increased sweat secretion and greater heat loss.

### 4.2. The predictive model including factors available for the whole process of labor

To establish model W, we gathered data on factors pertinent to the entire labor process, both before and after the administration of labor analgesia. Among the optimal predictors identified in model W, primiparity, NEUT count, anemia, and estimated fetal weight were consistent with those found in model B. New predictors for model W included the total duration of labor, the time from membrane rupture to delivery, and maternal height.

Research has shown that parturients with a longer duration of labor face an increased risk of fever, as supported by several studies.^[15,18,19]^ As previously noted, extended labor stages may lead to heightened inflammation levels. Furthermore, a prolonged interval since membrane rupture can elevate the risk of infection in parturients.^[20]^

### 4.3. Clinical value of the 2 predictive models

Model B, established in this study, could effectively predict the risk of intrapartum fever before labor analgesia. We believe this model is more suitable for use by anesthesiologists. According to the nomogram, the risk of fever for every parturient can be determined. Predictors such as body surface area, primiparity, NEUT count, anemia, and estimated fetal weight are difficult to control in clinical practice. However, the cervical dilatation degree is relatively easy to control before labor analgesia. For parturients with a high risk of fever, anesthesiologists can consult with obstetricians to determine whether to take measures to promote cervical dilation before providing labor analgesia. It is better for labor analgesia to be administered when the cervical dilatation degree is large. In addition, anesthesiologists can also take other measures; for example, steroid drugs can be used to reduce the incidence of fever in high-risk parturients.^[9]^

Although model B has the advantage of predicting fever earlier than model W, this study was unable to include predictors such as interleukin (IL) levels, which may be closely related to fever during labor, resulting in a weaker prediction efficacy of model B than model W. We believe that model W is more suitable for use by obstetricians. After the implementation of labor analgesia, obstetricians can use model W to further screen high-risk parturients. In model W, the total duration of labor and the time from the rupture of membranes to delivery can be altered through clinical interventions. Obstetricians can develop management strategies based on this model. Parturients with prolonged labor can take measures to accelerate the labor process, which can shorten the total labor process and the time from the rupture of membranes to delivery. In addition, if the time from the rupture of membranes to delivery is too long, obstetricians can use prophylactic antibiotics.

### 4.4. Limitations of the study

This study presents several limitations. Firstly, its retrospective design relies on existing medical records, which may introduce bias and limit causal inference, particularly as it was conducted at a single institution, affecting the generalizability of findings. Secondly, maternal temperature was measured every 2 hours using a mercury thermometer, which does not allow for continuous monitoring, potentially missing transient fever episodes. Thirdly, the exclusion of inflammatory biomarkers such as IL-6, IL-8, and IL-10, which could enhance predictive accuracy, also represents a limitation. Lastly, the specific clinical practices and demographics of the studied population may not reflect those in other healthcare settings, limiting the applicability of the results. Thus, further multicenter and prospective studies are warranted to validate the models and enhance their predictive capabilities.

## 5. Conclusion

This study successfully developed and validated 2 predictive models for assessing the risk of intrapartum fever. Model B identifies parturients at risk before labor analgesia, while model W evaluates risk throughout labor. These models enhance clinical decision-making, enabling early identification and intervention for high-risk patients, which may improve maternal and neonatal outcomes.

## Author contributions

**Conceptualization:** Bo Liu, Liang Ling, Yuanling Li, Fei Jia, Hongquan Xiao, Jian Zhang.

**Data curation:** Bo Liu, Liang Ling, Huiru Li, Na Li.

**Formal analysis:** Liang Ling, Dayuan Wei.

**Funding acquisition:** Na Li, Jian Zhang.

**Investigation:** Dayuan Wei, Hongquan Xiao.

**Methodology:** Liang Ling.

**Project administration:** Dayuan Wei.

**Resources:** Dayuan Wei.

**Supervision:** Dayuan Wei, Fei Jia, Hongquan Xiao.

**Validation:** Yuanling Li, Fei Jia.

**Visualization:** Yuanling Li, Huiru Li.

**Writing – original draft:** Bo Liu.

**Writing – review & editing:** Jian Zhang.

## Supplementary Material


