# Supplementary material for: Predictive models for intrapartum maternal fever: Development and validation of pre-analgesia and labor process indicators
Source: Medicine (Baltimore). 2025 Jun 20;104(25):e42939. doi: 10.1097/MD.0000000000042939 (PMC12187268; doi:10.1097/MD.0000000000042939)
Supplement: Supplementary file 1 [file medi-104-e42939-s001.docx]

**Table S1** Demographic and clinical characteristics of the development and validation cohorts.

| **Variable** | **Development cohort（n=2276）** | **Validation cohort（n=507）** | ***P*** |
| --- | --- | --- | --- |
| Age（y） | 28.58±3.74 | 28.91±3.69 | 0.069 |
| Height（cm） | 159.35±4.71 | 159.50±4.62 | 0.507 |
| Weight（kg） | 65.90±8.09 | 66.15±6.96 | 0.488 |
| BMI（kg/m^2^） | 25.95±2.92 | 26.01±2.64 | 0.648 |
| Body surface area（m^2^） | 1.79±0.12 | 1.79±0.11 | 0.442 |
| Gestational age（w） | 39.33±1.25 | 39.26±1.27 | 0.277 |
| WBC count（10^9^/L） | 9.16±2.46 | 8.98±2.12 | 0.097 |
| LYM count（10^9^/L） | 1.52±0.43 | 1.52±0.44 | 0.904 |
| LYM（%） | 17.35±5.42 | 17.60±5.86 | 0.353 |
| NEUT count（10^9^/L） | 6.92±2.29 | 6.75±2.01 | 0.098 |
| NEUT（%） | 74.63±6.34 | 74.42±6.99 | 0.502 |
| PLR（%） | 120.95±47.16 | 125.91±52.29 | 0.035 |
| NLR（%） | 4.92±2.26 | 4.91±2.44 | 0.973 |
| Estimated fetal weight（g） | 3205.21±369.36 | 3233.18±351.31 | 0.118 |
| Primiparity | 1860（81.7%） | 421（83.0%） | 0.523 |
| GDM | 526（23.1%） | 95（18.7%） | 0.034 |
| Pregnancy-induced hypertension | 90（4.0%） | 19（3.7%） | 0.408 |
| Anemia | 492（21.6%） | 129（25.4%） | 0.067 |
| Hepatitis B | 113（5.0%） | 31（6.1%） | 0.318 |
| Hypothyroidism | 137（6.0%） | 37（7.3%） | 0.310 |
| PROM | 71（36.2%） | 715（34.4%） | 0.639 |
| Cervical dilatation degree before labor analgesia | 1.41±0.90 | 1.49±0.87 | 0.063 |
| Number of vaginal examinations before labor analgesia | 2.47±1.24 | 2.33±1.22 | 0.021 |
| Oxytocin use before labor analgesia | 798（35.1%） | 170（33.5%） | 0.536 |
| Number of vaginal examinations^＃^ | 5.77±2.27 | 5.69±2.29 | 0.476 |
| Amniotic fluid pollution III^＃^ | 375（16.5%） | 69（13.6%） | 0.123 |
| Oxytocin^＃^ | 1126（49.5%） | 257（50.7%） | 0.624 |
| Duration of labor analgesia（min）^＃^ | 439.42±280.58 | 426.51±272.17 | 0.351 |
| Total duration of labor（min）^＃^ | 608.34±269.75 | 613.87±277.47 | 0.674 |
| Time from the rupture of membranes to delivery（min）^＃^ | 397.26±389.02 | 429.54±399.04 | 0.093 |

＃：The index of the whole labor and childbirth process
